# Supplementary material for: Effect of Sintering Temperature on Adhesion Property and Electrochemical Activity of Pt/YSZ Electrode
Source: Materials (Basel). 2022 May 12;15(10):3471. doi: 10.3390/ma15103471 (PMC9143013; doi:10.3390/ma15103471)
Supplement: Supplementary file 1 [file materials-15-03471-s001.zip › materials-1650547-supplementary.pdf]

materials

Article

Effect of Sintering Temperature on Adhesion Property and Electrochemical Activity of Pt/YSZ Electrode

Jixin Wang [1,2,3], Jiandong Cui [1,2,3], Xiao Zhang [1,2], Wentao Tang [1,2,3] and Changhui Mao [1,2,3,*]

1 GRINMAT State Key Laboratory of Advanced Materials for Smart Sensing, GRINM Group Co., Ltd., Beijing 100088, China; wangjixin68@163.com (J.W.); cuijiandong@grinm.com (J.C.); zhangxiao@grinm.com (X.Z.); youyantangwentao@126.com (W.T.)
2 GRIMAT Engineering Institute Co., Ltd., Beijing 101407, China
3 General Research Institute for Nonferrous Metals, Beijing 100088, China
* Correspondence: mao@grinm.com; Tel.: +86-1391-026-5812

The Supplementary Figures

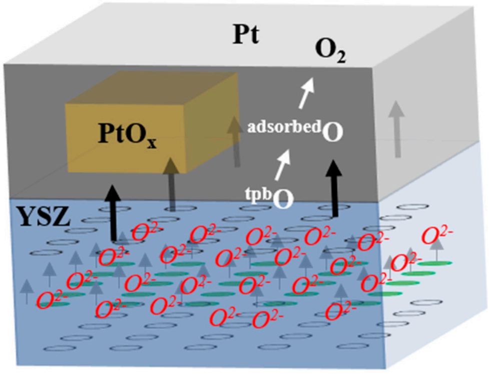

Figure S1. The possible anodic reaction model of the Pt/YSZ electrode [9].

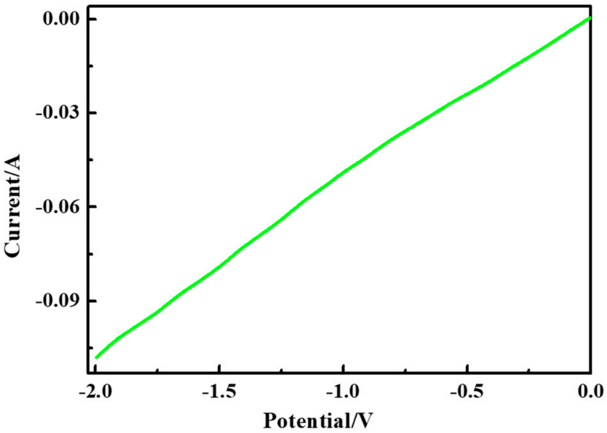

Figure S2. The LSV curve of the Pt/YSZ electrode when the scan voltage is expanded to -2 V.

Citation: Wang, J.; Cui, J.; Zhang, X.; Tang, W.; Mao, C. Effect of Sintering Temperature on Adhesion Property and Electrochemical Activity of Pt/YSZ Electrode. *Materials* **2022**, *15*, 3471. https://doi.org/10.3390/ma15103471

Academic Editor: Leszek Zaraska

Received: 8 March 2022
Accepted: 28 April 2022
Published: 12 May 2022

Publisher's Note: MDPI stays neutral with regard to jurisdictional claims in published maps and institutional affiliations.

Copyright: © 2022 by the authors. Licensee MDPI, Basel, Switzerland. This article is an open access article distributed under the terms and conditions of the Creative Commons Attribution (CC BY) license (https://creativecommons.org/licenses/by/4.0/).

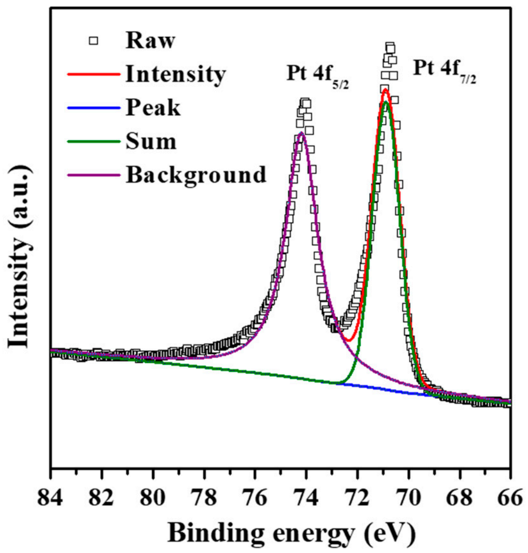

Figure S3. The photoelectron spectrum obtained from the surface of the Pt/YSZ electrode.
